# Fibrin-Based Scaffold Augmentation During Meniscal Repair May Enhance Healing and Functional Outcomes: A Systematic Review

**DOI:** 10.1177/03635465261440395

**Published:** 2026-06-03

**Authors:** Khaled Skaik, Harjind Kahlon, Marc Daniel Bouchard, Prushoth Vivekanantha, Etienne L. Belzile, Bogdan A. Matache, Jihad Abouali

**Affiliations:** * Faculty of Medicine and Health Sciences, McGill University, Montreal, Quebec, Canada; † Temerty faculty of Medicine, University of Toronto, Toronto, Ontario, Canada; ‡ Division of Orthopaedic Surgery, Department of Surgery, McMaster University, Hamilton, Ontario, Canada; ‖ Department of Surgery, Centre Hospitalier Universitaire de Quebec, Laval University, Quebec City, Quebec, Canada; ¶ Division of Orthopaedic Surgery, Faculty of Medicine, University of Ottawa, Ottawa, Ontario, Canada; # Division of Orthopaedic Surgery, Department of Surgery, University of Toronto, Toronto, Ontario, Canada; Investigation performed at McMaster University, Hamilton, Ontario, Canada

**Keywords:** meniscus, meniscal repair, fibrin, augmentation

## Abstract

**Background:**

Biologic augmentation techniques, such as fibrin-based scaffolds, have been proposed to enhance healing of meniscal tears, particularly in avascular zones. However, clinical outcomes remain variably reported.

**Study Design:**

Systematic review; Level of evidence, 4.

**Purpose:**

To evaluate the healing rate, complication rate, and patient-reported outcome scores after meniscal repair augmented with fibrin-based scaffolds.

**Methods:**

A systematic review was conducted according to PRISMA (Preferred Reporting Items for Systematic Reviews and Meta-Analyses) guidelines, searching PubMed, Embase, and Ovid MEDLINE through October 15, 2025. Studies evaluating fibrin-based augmentation during meniscal repair were included. Primary outcomes were healing and complication rates. Secondary outcomes included International Knee Documentation Committee (IKDC), Lysholm, and Tegner scores.

**Results:**

Of 757 studies, 15 met inclusion criteria, comprising 438 patients with meniscal tears in mostly avascular zones. Of these, 345 underwent fibrin-based augmentation; the rest did not. There were 4 fibrin-based methods identified: fibrin clot from peripheral venous blood (FC-PVB; n = 252), fibrin clot from bone marrow aspirate concentrate (FC-BMAC; n = 30), platelet-rich fibrin (PRF; n = 15), and PRF combined with platelet-rich plasma (PRF+PRP; n = 48). Among patients who received FC-PVB, 77.5% achieved complete healing, 12.2% partial healing, and 10.3% failure. Complications occurred in 8.2% of patients who received FC-PVB and included paresthesia and reinjuries; 2.6% reported persistent knee pain. In FC-PVB studies, significant improvements were seen in IKDC and Lysholm scores but not in Tegner scores. Other fibrin-based methods showed consistent improvements across all scores. Compared to nonaugmented repair, fibrin-based augmentation yielded superior outcomes.

**Conclusion:**

Fibrin-based augmentation demonstrated high healing rates, low complication rates, and improved clinical outcome scores. Compared to nonaugmented repair, fibrin-based scaffolds achieved better healing and function. FC-PVB enhanced symptom relief and functional recovery, while only FC-BMAC showed improvements in activity levels.

Meniscal injuries have been associated with increased contact pressure in the knee joint, potentially leading to cartilage damage and progressive degeneration of the involved compartment.^8,42^ While debridement of meniscal tears can help relieve mechanical symptoms, it does not restore native joint biomechanics. As a result, contemporary literature increasingly advocates for meniscal preservation whenever feasible.^50^ Repairing meniscal tears has been shown to help restore normal contact mechanics, decelerate joint degeneration, and lower the likelihood of requiring joint replacement in the future.^67^

The outcome of meniscal repair is influenced by various patient- and surgery-related factors.^27^ A careful intraoperative assessment of the tear's location, morphology, and extent is essential to determine its reparability. This is largely because of limited vascularity of the meniscus, as the perimeniscal capillary plexus supplies only the outer 10% to 25% of tissue and serves as the sole source of fibrocartilaginous healing.^3^ Other characteristics including tear chronicity, complexity, degree of meniscal degeneration, and meniscal instability all affect the capacity for meniscal healing.^58,62^ In addition, tear morphology varies depending on the mechanism.^58^ Rotational forces typically produce vertical or bucket-handle tears, radial tears disrupt circumferential hoop stress transmission, root tears detach the meniscus from its tibial insertion, and chronic degenerative loading more often results in horizontal cleavage tears.^58^

Meniscal repair may be performed using different approaches.^59^ Inside-out repair remains a dependable option for posterior horn and midbody tears because of strong suture fixation, whereas outside-in techniques are commonly preferred for anterior horn lesions.^59^ All-inside devices provide less invasive fixation with reduced soft tissue dissection while demonstrating comparable biomechanical strength in appropriate tear patterns.^59^

Numerous biologic augmentation strategies have been proposed in the literature especially for tears that are at a high risk of failure, including chronic, radial, complex, and horizontal cleavage tears. These include platelet-rich plasma (PRP),^28,65^ marrow stimulation techniques,^16,25^ mesenchymal stem cells (MSCs),^23,24,56^ and fibrin-based approaches.^4,66^

While PRP, MSCs, and marrow stimulation techniques have demonstrated promising results, their use is often limited by technical complexity, procedural invasiveness, prolonged operative time, and high cost. In contrast, fibrin clots offer a practical alternative. They are associated with a lower cost and are simpler to prepare, relying on the intrinsic coagulation of the patient's venous blood with no need for specialized processing.^6,27^

The use of fibrin to enhance the healing of high-risk meniscal tears was first described over 3 decades ago.^21^ Fibrin-based augmentation can be achieved through multiple autologous sources, including a fibrin clot derived from the patient's peripheral venous blood (FC-PVB),^9,12,15,22,33^ a fibrin clot derived from bone marrow aspirate concentrate (FC-BMAC),^20^ or platelet-rich fibrin (PRF).^36,37,55^

This systematic review aimed to evaluate the healing rate, complication rate, and patient-reported outcome scores after meniscal repair using fibrin-based scaffolds. It was hypothesized that fibrin-based augmentation in meniscal repair would be associated with high healing rates, improved patient-reported outcome scores, and low complication rates.

## Methods

### Study Design

This review adhered to PRISMA (Preferred Reporting Items for Systematic Reviews and Meta-Analyses) guidelines.^51^ A comprehensive search was performed in Embase, PubMed, and Ovid MEDLINE from database inception to October 15, 2025. The detailed search strategy is available in Appendix 1 (available in the online version of this article). There were 2 independent reviewers (K.S. and H.K.) who screened articles using Covidence software (Veritas Health Innovation). Any disagreements were resolved with a third author (M.D.B.), and data were extracted using Google Sheets.

### Eligibility Criteria

Studies of any design were included if they evaluated the use of fibrin-based scaffolds for the treatment of meniscal tears and reported healing outcomes. Studies that included isolated meniscal repair as well as meniscal repair performed concomitantly with anterior cruciate ligament (ACL) reconstruction (ACLR) were eligible for inclusion. However, outcomes from the 2 groups were reported separately. Studies including meniscal tears of any type, except root tears, were included. Importantly, outcomes were stratified by tear type, zone, and location when available. Studies involving inflammatory or rheumatoid arthritis, infections, multiligamentous injuries, or lower limb malalignment were excluded. Studies in which meniscal tears were associated with injuries other than ACL tears were also excluded. Studies on revision meniscal repair were excluded. Additionally, studies that involved meniscal transplantation and not repair of the native meniscus were excluded. Each study was assessed on whether it included an overlapping patient population from another publication and whether it reported the same outcome. Studies that described surgical techniques without reporting clinical results were excluded. In cases in which a duplicate patient population was clearly described across multiple publications, only the most recent study was included.

### Quality Assessments

Case series and case reports were assessed using the Joanna Briggs Institute (JBI) critical appraisal checklist,^45^ and the Modified Coleman Methodology Score (MCMS) was employed for observational and randomized studies, as done in other reviews.^30,45,60,63^ The JBI critical appraisal checklist was used according to the instructions provided by the institution and consisted of 10 items rating the quality of the study.^45^ A higher score corresponded with better quality studies, and ideally, each article would achieve 10 points. The MCMS is a tool with 10 categories with a maximum of 100 points, with a higher score generally indicating minimized confounding factors and a low risk of bias.^30^

### Outcomes of Interest

The primary outcomes were the healing rate and complication rate. Complete healing was defined as the restoration of meniscal tissue continuity with no evidence of a residual cleft, a defect, or instability on imaging (magnetic resonance imaging [MRI] or second-look arthroscopy), accompanied by the resolution of symptoms and no mechanical signs.^43^ Partial healing was defined as incomplete repair of the meniscal tear with an improved but not full restoration of mechanical integrity. Secondary outcomes included International Knee Documentation Committee (IKDC), Lysholm, and Tegner scores.

### Statistical Analysis

Descriptive statistics were used to report data. Interreviewer agreement during screening was assessed using the kappa (κ) statistic. Agreement was defined as follows: 0.91-0.99, almost perfect agreement; 0.71-0.90, substantial agreement; 0.61-0.70, high agreement; 0.41-0.60, moderate agreement; 0.21-0.40, fair agreement; and ≤0.20, no agreement.^38^

## Results

### Study Characteristics

Of 757 studies, 64 underwent a full-text review, and 15 studies met the inclusion criteria (Figure 1). Interreviewer agreement was substantial during the title and abstract screening stage (κ = 0.83 [95% CI, 0.84-0.87]) and almost perfect during the full-text review stage (κ = 0.94 [95% CI, 0.91-0.96]). Overall, 9 studies were prospective case series, 2 were retrospective case series,^15,20^, 1 was a randomized prospective study^9^, 1 was prospective cohort study,^37^ 1 was a retrospective cohort study,^55^ and 1 was a randomized controlled trial.^12^

**Figure 1.**
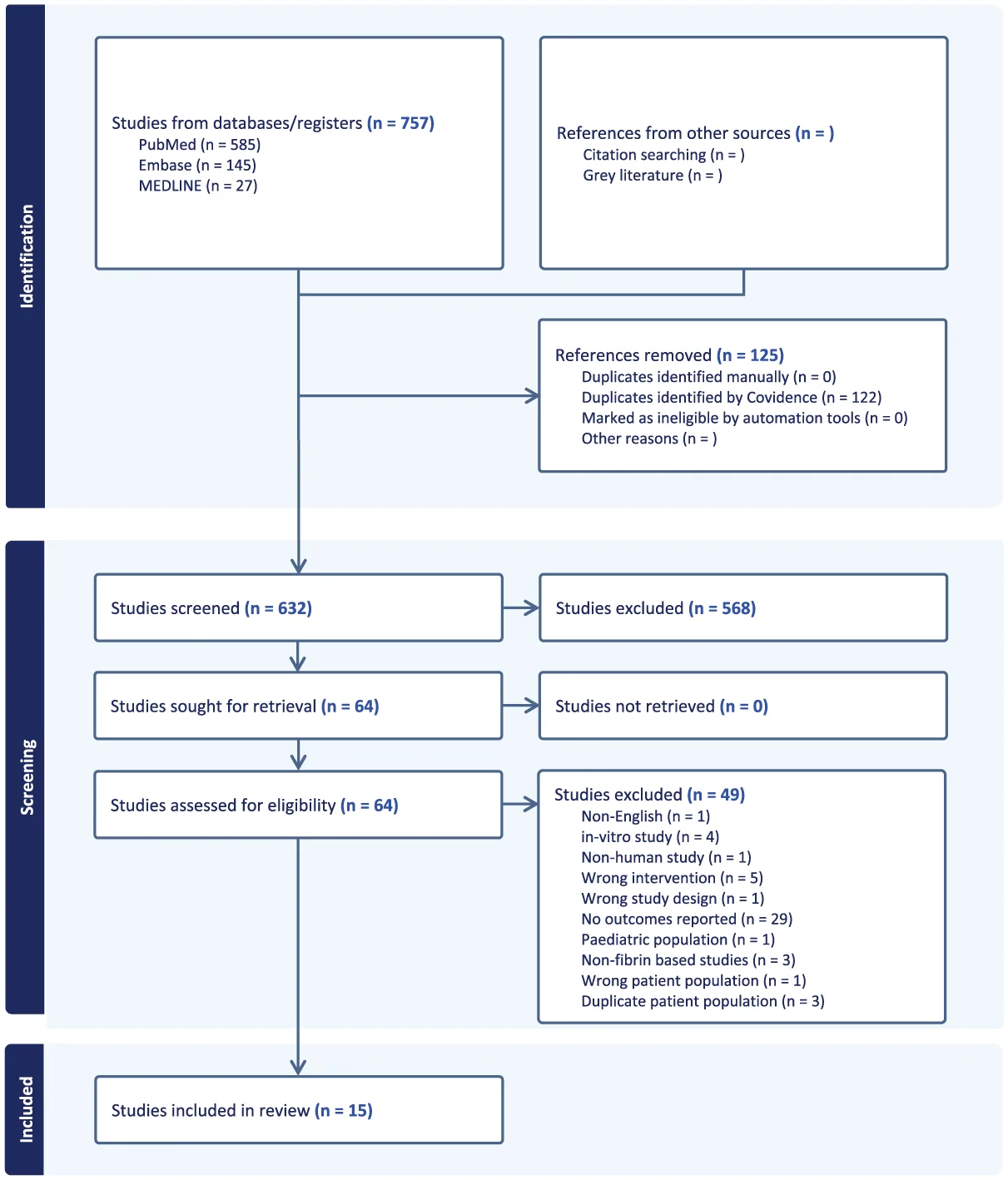
PRISMA (Preferred Reporting Items for Systematic Reviews and Meta-Analyses) flowchart of included studies.

### Study Quality

The mean JBI score was 8.7 (range, 7-10), and the mean MCMS score was 70.5 (range, 55-83), across included studies (Appendix 2 [available online]).

### Patient Characteristics

A total of 438 patients with 461 menisci were included, of which 345 underwent fibrin-based augmentation during meniscal repair; the rest did not undergo augmentation. Of all tears, 220 were in the medial meniscus, 180 were in the lateral meniscus, and 61 were not specified. Across tear types, 122 were horizontal, 68 radial, 53 bucket-handle, 73 longitudinal, 26 vertical, 1 flap, and 82 complex tears. These tears were mainly located in either the red-white zone or white-white zone, with only 5 studies reporting some tears in the red-red zone^15,29,33,46,53^ (Table 1). The mean age of patients ranged from 20 to 47 years. There were 4 studies that included a subset of patients who underwent concomitant ACLR with augmented meniscal repair, comprising 72 patients of the overall cohort included in this review.^22,29,33,66^ Another concomitant procedure included 1 case of meniscal cyst decompression.^66^ The mean time from the meniscal injury to repair ranged from 1.5 to 25.4 months (Table 1).

**Table 1:** Study and Patient Characteristics*^*a*^*

First Author (Year)	Study Design (Level of Evidence)	Type of Augmentation	No. of Patients	Female Sex, %	Age,*^*b*^* y	Location of Meniscal Tear, n	Zone and Type of Meniscal Tear, n	Follow-up,*^*b*^* mo	Concomitant Surgery, n (%)	Time From Injury to Surgery*^*b*^*
Biedert^9^ (2000)	Randomized prospective study (3)	FC-PVB	FC-PVB: 7 Non–FC-PVB: 33	47.5	30.4 (16.0-50.0)	FC-PVB: 7 MM Non–FC-PVB: 33 MM	FC-PVB: 7 WW (7 horizontal grade 2) Non–FC-PVB: 33 WW (33 horizontal grade 2)	26.5 (12.0-38.0)	None	—
Chrysanthou^12^ (2024)	Randomized controlled trial (2)	FC-PVB	FC-PVB: 24 Non–FC-PVB: 27	13.7	28.2 (30.0-49.0)	FC-PVB: 20 MM, 4 LM (16 medial third, 8 posterior third) Non–FC-PVB: 22 MM, 5 LM (20 medial third, 7 posterior third)	FC-PVB: 18 RW, 6 WW (8 radial, 8 bucket handle, 5 longitudinal, 3 horizontal) Non–FC-PVB: 22 RW, 5 WW (9 radial, 9 bucket handle, 6 longitudinal, 3 horizontal)	3.0, 12.0	None	—
Davies^15^ (2024)	Retrospective case series (4)	FC-PVB	51	37.2	34.0 (14.0-60.0)	32 MM, 19 LM (24 posterior horn, 14 posterior horn–body, 7 body, 1 anterior horn–body, 3 anterior horn, 2 complete)	3 RR, 12 RW, 36 WW (16 radial, 8 vertical, 7 horizontal, 7 complex nonradial, 13 complex radial)	46.0 (22.0-87.0)	None	122 d
Hashimoto^20^ (2022)	Retrospective case series (4)	FC-BMAC	30	33.3	23.2 (17.0-42.0)	6 MM, 24 LM (23 discoid)	30 WW (2 radial, 1 flap, 21 horizontal, 6 complex)	25.9 (24.0-48.0)	None	—
Henning^22^ (1991)	Prospective case series (4)	FC-PVB	31	—	—	31 (not specified)	Not specified (31 complex)	6-9	ACLR: 25 (80.6)	—
Ishimura^29^ (1997)	Prospective case series (4)	FC-PVB	40	67.5	20.0 (12.0-45.0)	28 MM, 33 LM	44 RR, 11 RW, 6 WW (11 bucket handle, 50 longitudinal)	98.4 (122.4-136.8)	ACLR: 30 (75.0)	—
Kale^33^ (2022)	Prospective case series (4)	FC-PVB	30	—	—	30 (not specified)	30 RR (6 radial, 9 bucket handle, 7 longitudinal, 8 horizontal)	3.0, 6.0, 9.0, 12.0, 24.0	ACLR: 16 (53.3)	—
Kamimura^34^ (2014)	Prospective case series (4)	FC-PVB	10	50.0	35.8 (15.0-63.0)	4 MM, 6 LM (3 lateral discoid)	Not specified (10 horizontal)	40.8 (32.5-51.3)	None	5.6 mo
Kemmochi^36^ (2024)	Prospective case series (4)	PRF+PRP	31	38.7	39.1 (15.0-69.0)	16 MM, 15 LM (12 anterior, 19 middle, 24 posterior (tears spanning multiple segments counted once per segment))	Not specified (18 vertical, 21 horizontal, 3 radial, 13 complex) (tears reported per segment-level rupture site, not per distinct tear type)	24.0	None	—
Kemmochi^37^ (2018)	Prospective cohort study (3)	PRF+PRP	PRF+PRP: 17 Non–PRF+PRP: 5	PRF+PRP: 47.1 Non–PRF+PRP: 20.0	PRF+PRP: 32.4 (15.0-69.0) Non–PRF+PRP: 20.8 (13.0-38.0)	PRF+PRP: 6 MM, 13 LM (5 anterior, 2 middle, 6 posterior, 1 anterior-posterior, 3 middle-posterior, 2 anterior-middle-posterior) Non–PRF+PRP: 1 MM, 4 LM (2 anterior, 2 posterior, 1 anterior-middle-posterior)	PRF+PRP: not specified (19 horizontal ) Non–PRF+PRP: not specified (5 horizontal)	6.0	None	PRF+PRP: 60.9 d Non–PRF+PRP: 46.0 d
Nakayama^46^ (2020)	Prospective case series (4)	FC-PVB	24	16.7	47.0 (35.0-66.0)	24 MM	RR or RW (predominantly horizontal [frequency not specified])	39.3 (24.0-64.0)	None	—
Nakayama^47^ (2017)	Prospective case series (4)	FC-PVB	FC-PVB:18 Non-FC-PVB : 28	26.0	22.9 (12.0-50.0)	FC-PVB:11 MM and 7 LM. Non-FC_PVB: 7 MM, 21 LM. Total : 18 MM (50% middle-to-posterior horn), 28 LM (30% middle-to-posterior horn)	RW and WW (MM: 4 longitudinal, 1 radial, 5 complex, 8 bucket handle; LM: 1 longitudinal, 4 radial, 3 complex, 8 bucket handle, 12 unspecified)	19.8 (12.0-30.0)	None	—
Ra^53^ (2013)	Prospective case series (4)	FC-PVB	12	8.3	—	3 MM, 9 LM	RW and RR (12 radial)	30.0 (22.0-38.0)	None	—
Screpis^55^ (2021)	Retrospective cohort study (3)	PRF	15	47.0	32.8 (21.0-53.0)	15 LM (1 anterior horn, 14 posterior horn–body)	Not specified (9 horizontal, 4 complex, 2 radial)	42.6 (33.0-58.0)	—	—
van Trommel^66^ (1998)	Prospective case series (4)	FC-PVB	5	—	20.0 (18.0-22.0)	5 LM (posterolateral)	WW (5 complete radial)	71.0 (66.0-81.0)	ACLR: 1 (20.0) Meniscal cyst decompression: 1 (20.0)	25.4 mo

a ACLR, anterior cruciate ligament reconstruction; FC-BMAC, fibrin clot from bone marrow aspirate concentrate; FC-PVB, fibrin clot from peripheral venous blood; LM, lateral meniscus; MM, medial meniscus; PRF, platelet-rich fibrin; PRP, platelet-rich plasma; RR, red-red; RW, red-white; WW, white-white.

b Data are shown as mean, range, or mean (range).

### Fibrin-Based Augmentation

A total of 11 studies reported using FC-PVB, encompassing 252 patients.^9,12,15,22,29,33,34,46,47,53,66^ One study used FC-BMAC harvested from the intercondylar notch of the knee joint in 30 patients.^20^ Additionally, 2 studies used PRF+PRP, both derived from autologous PVB^36,37^; PRF membranes were inserted in the meniscal cleft, and PRP was subsequently injected in 48 patients.^36,37^ One study utilized PRF prepared from autologous blood using RegenKit Extracell (Regen Lab), producing a PRF matrix that was sutured to the capsule as a biologic scaffold in 15 patients.^55^

### Healing

A total of 9 studies reported the healing status of 213 menisci treated with FC-PVB.^12,22,29,33,34,46,47,53,66^ Among these, 116 menisci were from patients in studies that included a subset that underwent concomitant ACLR (Table 2). Healing status was assessed by MRI in 3 studies,^12,33,46^ by second-look arthroscopy in 3 studies,^22,29,34^ and by both MRI and arthroscopy in the remaining 3 studies.^47,53,66^

**Table 2:** Healing Rates*^*a*^*

First Author (Year)	Zone of Meniscal Tear	No. of Menisci	Evaluation of Healing Status	Follow-up,*^*b*^* mo	Complete Healing, n (%)	Partial Healing, n (%)	Failed Healing, n (%)
Chrysanthou^12^ (2024)	FC-PVB: 18 RW, 6 WW Non–FC-PVB: 22 RW, 5 WW	FC-PVB: 24 Non– FC-PVB: 27	MRI	12.0	FC-PVB: 22 (91.7) Non–FC-PVB: 19 (70.4)	FC-PVB: 1 (4.2) Non–FC-PVB: 7 (25.9)	FC-PVB: 1 (4.2) Non–FC-PVB: 1 (3.7)
Henning^22^ (1991)	Not specified	31	Arthroscopy	6-9	10 (32.3)	16 (51.6)	5 (16.1) (2 radial split, 1 flap, 1 double longitudinal, 1 bucket handle)
Ishimura^29^ (1997)	20 RR, 7 RW, 4 WW	31	Arthroscopy	10.2 (2-35)	23 (74.2) (12 MM, 11 LM; 17 RR, 5 RW, 1 WW; 16 longitudinal, 7 bucket handle)	3 (9.7) (1 MM, 2 LM; 1 RR, 1 RW, 1 WW; 3 longitudinal)	5 (16.1) (1 MM, 4 LM; 2 RR, 1 RW, 2 WW; 4 longitudinal, 1 bucket handle)
Kale^33^ (2022)	30 RR	30	MRI	24.0	29 (96.7) (all RR)	—	1 (3.3) (RR; complex)
Kamimura^34^ (2014)	Not specified	10	Arthroscopy	6.7 (5.3-7.5)	7 (70.0) (2 MM, 2 LM, 3 lateral discoid; all horizontal cleavage)	3 (30.0) (2 MM, 1 LM; all horizontal cleavage)	—
Nakayama^46^ (2020)	24 RR or RW	24	MRI	39.3 (24-64)	18 (75.0) (18 MM; mostly complex horizontal)	—	6 (25.0) (6 MM; mostly complex horizontal)
Nakayama^47^ (2017)	46 RW and WW	46	Arthroscopy and MRI	19.8 (12-30)	42 (91.3) (16 MM, 26 LM)	—	4 (8.7) (2 MM, 2 LM; 3 bucket handle, 1 not specified)
Ra^53^ (2013)	RW and RR (frequency not specified)	12	12 MRI, 7 arthroscopy	MRI: 11 Arthroscopy: 20	11 (91.7) (11 MRI, 6 arthroscopy; ; complete radial)	1 (8.3) (MRI and arthroscopy; complete radial)	—
van Trommel^66^ (1998)	5 WW	5	5 arthroscopy, 3 MRI	Arthroscopy: 4 (3-6) MRI: 71.0 (66-81)	3 (60.0) (3 MRI, 3 arthroscopy; 3 LM; 3 WW; complete radial)	2 (40.0) (2 arthroscopy; 2 LM; 2 WW; complete radial)	—
Total, n (%)	—	213 (100.0)	—	—	165 (77.5)	26 (12.2)	22 (10.3)

a FC-PVB, fibrin clot from peripheral venous blood; LM, lateral meniscus; MM, medial meniscus; MRI, magnetic resonance imaging; RR, red-red; RW, red-white; WW, white-white.

b Data are shown as mean, range, or mean (range).

Of the 9 studies, 6 explicitly defined the criteria for complete, partial, and incomplete/failed healing.^22,29,34,46,53,66^ Among arthroscopy-based studies, 1 study defined healing based on the percentage of the vertical tear height restored (>90% complete, 50%-90% incomplete, <50% failure),^22^ whereas 3 others defined complete healing as full tear closure with firm adhesion and tissue continuity^29,34,47^ and partial healing as incomplete closure or a residual cleft.^29,34^ Among MRI-based studies, healing was consistently defined as the absence of persistent intrameniscal fluid signaling throughout the repair site, and the presence of some fluid signaling extending through the meniscus was considered as partial healing.^46,53,66^

At a minimum follow-up of 2 months, 165 menisci (77.5%) were completely healed, 26 (12.2%) were partially healed, and 22 (10.3%) were unhealed or had failed repair (Table 2). When restricting the analysis to only those studies reporting outcomes exclusively from isolated meniscal repair, 100 menisci (86.2%) were completely healed, 5 (4.3%) were partially healed, and 11 (9.5%) failed to heal (Table 2). Compared to the non–fibrin clot (NFC) group, one study demonstrated that the complete healing rate was significantly higher in the FC-PVB group (70.4% vs 91.7%, respectively; *P* < .05).^12^ The healing rate was not reported by studies that utilized PRF, PRF+PRP, or FC-BMAC.^20,36,37,55^

### Knee Pain and Complications

A total of 8 studies reported complications.^9,12,15,20,29,33,34,47^ Among FC-PVB studies, 22 of 268 patients (8.2%) had complications including paresthesia, effusion, and reinjuries, requiring implant removal or further debridement in some cases (Table 3). In addition, knee pain was reported in 2.6% of patients^9,33^ (Table 3). When limiting the analysis to studies that reported outcomes exclusively from isolated meniscal repair, 18 of 228 patients (7.9%) experienced complications (Table 3). There were 5 patients in the FC-BMAC group who had complications of positive Barrett criteria,^7,20^ joint line tenderness, or knee effusion.^20^ No complications were reported in menisci augmented by PRF+PRP or PRF alone (Table 3).

**Table 3:** Rates of Complications and Knee Pain*^*a*^*

First Author (Year)	Type of Augmentation	Location and Zone of Meniscal Tear, n	Type of Meniscal Tear, n	Complications, n (%)	Knee Pain, n (%)
Biedert^9^ (2000)	FC-PVB	All MM (zone not specified)	Horizontal	FC-PVB: 1 (14.3) saphenous nerve paresthesia Non–FC-PVB: 1 (3.0) saphenous nerve paresthesia	FC-PVB: 6 (85.7) Non–FC-PVB: 4 (12.1)
Chrysanthou^12^ (2024)	FC-PVB	Not specified	Not specified	FC-PVB: 2 (8.3) positive McMurray/Apley test result Non–FC-PVB: 3 (11.1) positive McMurray/Apley test result	—
Davies^15^ (2024)	FC-PVB	4 MM, 1 LM (3 WW, 1 RW, 1 RR)	2 radial, 2 vertical, 1 complex radial	5 (9.8) further repair/debridement needed	—
Hashimoto^20^ (2022)	FC-BMAC	2 MM, 3 lateral discoid(5 WW)	3 horizontal, 1 radial, 1 complex	3 (10.0) positive Barrett criteria (2 (6.7) joint line tenderness, 1 (3.3) effusion), 2 retears	—
Ishimura^29^ (1997)	FC-PVB	4 MM, 2 LM (4 RR, 1 RW, 1 WW) (4 concomitant ACL lesion [2 RR, 1 RW, 1 WW])	3 bucket handle, 3 longitudinal	6/40 patients (15.0) with meniscal symptoms, 6/61 menisci (9.8) with recurrent tear	—
Kale^33^ (2022)	FC-PVB	Not specified	1 complex with effusion, 2 not specified	2 (6.7) paresthesia, 1 (3.3) effusion	1 (3.3)
Kamimura^34^ (2014)	FC-PVB	Not specified	1 horizontal	1 (10.0) implant removal because of movement from original placement	—
Nakayama^47^ (2017)	FC-PVB	2 MM, 2 LM (zone not specified)	3 bucket handle, 1 not specified	4 (8.7) retear	—

a ACL, anterior cruciate ligament; FC-BMAC, fibrin clot from bone marrow aspirate concentrate; FC-PVB, fibrin clot from peripheral venous blood; LM, lateral meniscus; MM, medial meniscus; RR, red-red; RW, red-white; WW, white-white.

### IKDC Score

A total of 5 studies reported clinical subjective outcomes using the IKDC form across fibrin-based methods, none of which included patients who underwent concomitant ACLR.^34,36,37,53,55^ Among the FC-PVB studies, 2 reported IKDC scores and demonstrated statistically significant improvements from preoperatively to postoperatively, with the follow-up period ranging from 22 to 51.3 months (*P* < .05)^34,53^ (Table 4).

**Table 4:** Patient-Reported Outcome Scores*^*a*^*

First Author (Year)	Type of Augmentation	IKDC Score		Tegner Score		Lysholm Score	Tegner-Lysholm Score		
		Preoperative	Postoperative	Preoperative	Postoperative	Preoperative	Postoperative	Preoperative	Postoperative
Hashimoto*^20^* (2022)	FC-BMAC	—	—	6.1 ± 2.2	5.8 ± 2.0*^*b*^*	65.1 ± 11.7	95.6 ± 6.8*^*b*^*	—	—
Kamimura^34^ (2014)	FC-PVB	26.5 ± 19.0	87.8 ± 7.5*^*b*^*	—	—	69.3 ± 16.3	95.4 ± 3.6*^*b*^*	—	—
Davies^15^ (2024)	FC-PVB	—	—	4.0 (0.0-9.0)	5.0 (1.0-10.0)	59.0 (20.0-82.0)	95.0 (75.0-100.0)*^*b*^*	—	—
Chrysanthou^12^ (2024)	FC-PVB	—	—	—	—	—	—	63.0 ± 4.0	89.0 ± 5.0*^*b*^*
Kale^33^ (2022)	FC-PVB	—	—	—	—	67.6 ± 6.6	92.0 ± 2.9*^*c*^*	—	—
Nakayama^46^ (2020)	FC-PVB	—	—	—	—	75.4 ± 4.6	90.8 ± 5.7*^*b*^*	—	—
Nakayama^47^ (2017)	FC-PVB	—	—	MM: 6.1 ± 1.6 LM: 6.3 ± 2.0	MM: 5.7 ± 1.7 LM: 6.8 ± 2.0	MM: 74.0 ± 14.3 LM: 77.0 ± 15.7	MM: 98.3 ± 2.2*^*b*^* LM: 96.1 ± 5.0*^*b*^*	—	—
Ra^53^ (2013)	FC-PVB	57.0 ± 7.0	92.0 ± 3.0*^*c*^*	—	—	65.0 ± 6.0	94.0 ± 3.0*^*c*^*	—	—
Screpis^55^ (2021)	PRF	37.6 ± 5.1	89.4 ± 2.6*^*b*^*	—	—	—	—	41.3 ± 5.4	92.3 ± 4.6*^*b*^*
Kemmochi^37^ (2018)	PRF+PRP	28.9 ± 11.6	87.4 ± 10.4*^*b*^*	—	—	—	—	61.3 ± 11.1	95.8 ± 7.1*^*b*^*
Kemmochi^36^ (2024)	PRF+PRP	39.3 ± 19.0	6 mo: 84.0 ± 14.2*^*c*^* 12 mo: 91.0 ± 10.8*^*b*^* 24 mo: 90.4 ± 11.3*^*b*^*	5.4 ± 2.3	6 mo: 5.4 ± 2.1 12 mo: 5.6 ± 2.0 24 mo: 5.8 ± 2.0	59.7 ± 15.8	6 mo: 95.1 ± 7.2*^*b*^* 12 mo: 96.9 ± 6.1*^*b*^* 24 mo: 95.1 ± 7.8*^*b*^*	—	—

a Data are shown as mean ± SD or median (range). FC-BMAC, fibrin clot from bone marrow aspirate concentrate; FC-PVB, fibrin clot from peripheral venous blood; IKDC, International Knee Documentation Committee; LM, lateral meniscus; MM, medial meniscus; PRF, platelet-rich fibrin; PRP, platelet-rich plasma.

b *P* < .01.

c *P* < .05.

In PRF+PRP studies, IKDC scores significantly improved at 6 months’ follow-up and plateaued by 12 months postoperatively (*P* < .01).^36,37^ PRF augmentation also showed a significant improvement from preoperatively to postoperatively at a mean follow-up of 42.6 months (*P* < .01)^55^ (Table 4). Compared to NFC repair, one study showed that partial meniscectomy produced significantly better IKDC scores than both nonoperative management (*P* = .006) and fibrin clot repair (*P* = .003).^9^

### Tegner Score

A total of 4 studies reported Tegner activity scores at a follow-up interval ranging from 6 to 64 months, none of which included patients who underwent concomitant ACLR.^15,20,36,47^ Among FC-PVB studies, none demonstrated a statistically significant improvement in Tegner scores from preoperatively to postoperatively.^15,36,47^ One study using FC-BMAC reported a significant improvement from baseline to final follow-up (*P* < .01)^20^ (Table 4).

### Lysholm Score

A total of 8 studies reported Lysholm scores evaluated from preoperatively to postoperatively, with the follow-up period ranging from 3 to 39 months.^15,20,33,34,36,46,47,53^ Among the FC-PVB studies, 6 reported Lysholm scores,^15,33,34,46,47,53^ including 1 study^33^ that involved a subset of patients who underwent concomitant ACLR. All 6 studies demonstrated statistically significant improvements in Lysholm scores from baseline to final follow-up (*P* < .05).^15,33,34,46,47,53^ Similarly, FC-BMAC–augmented repair showed significant postoperative improvements in the Lysholm score (*P* < .01).^20^ The same observation was seen in PRF+PRP–augmented repair (*P* < .01).^36^ There were 3 studies that reported combined Tegner-Lysholm scores, all of which showed significant improvements from preoperatively to postoperatively (*P* < .01).^12,37,55^ Compared to NFC augmentation, one study reported greater improvements in Tegner-Lysholm scores with fibrin clot augmentation at 12 months (*P* < .001).^12^

## Discussion

The primary findings of this study indicate that meniscal repair augmented with FC-PVB resulted in 77.5% complete healing, 12.2% partial healing, and 10.3% failure. Complications were reported in 8.2% of patients in the FC-PVB group, with persistent knee pain documented in 2.8% of cases. The FC-PVB studies showed significant improvements in IKDC, Lysholm, and combined Tegner-Lysholm scores but not in Tegner scores. FC-BMAC augmentation improved Tegner and Lysholm scores, while PRF or PRF+PRP augmentation improved IKDC, Lysholm, and combined Tegner-Lysholm scores. Compared to NFC repair, superior outcomes were reported in healing rates and combined Tegner-Lysholm scores after fibrin clot repair, but IKDC scores were inconsistent.

This study showed that healing rates after the use of fibrin-based scaffolds were high and that only few cases did not heal at ≥12 months’ follow-up.This might be explained by the fact that fibrin-based scaffolds provide sustained benefits because of their slow polymerization, hemostatic properties, higher concentration of healing factors, a platelet density approximately 10 times greater than baseline, and support for cytokine activity and cellular migration.^44^ Serving as a biologic scaffold, the fibrin clot helps preserve the elongated morphology of meniscal cells, which is critical for tissue regeneration.^52,61^ Over time, the fibrin clot transitions from fibroconnective to fibrocartilaginous tissue, as growth factors and reparative cells are drawn to the site through chemotactic signals and mitotic activity is stimulated in the surrounding area.^53^ This sequential reparative cascade facilitates and accelerates meniscal healing. Notably, placing the fibrin clot into the repair site before knot tying leads to a more secure, water-tight closure, unlike marrow venting alone in which clot formation occurs after suturing.^52,61^

However, it is important to note that there are many factors that could influence healing of the meniscus, regardless of the augmentation technique.^5^ For instance, factors favoring healing include tears in the red-red zone, fresh tears, and radial tears, whereas those not favoring repair include complex, old, and degenerative tears as well as plastic deformation of tissue.^26,64^ In this review, few studies stratified healing outcomes by tear type,^29,34,53,66^ which limits the ability to draw definitive conclusions about which tear morphology derives the greatest healing benefit from fibrin-based augmentation.

Notably, in FC-PVB studies, IKDC and Lysholm scores improved significantly from preoperatively to postoperatively, whereas the Tegner score did not. Furthermore, when Lysholm and Tegner scores were combined, the composite score reached significance. This could be because the improvement was primarily driven by the Lysholm component rather than the Tegner component. One possible explanation is that the Tegner activity score reflects an actual return to high-level sport or strenuous activity, which may lag behind improvements in symptoms and function captured by the IKDC or Lysholm score; thus, the patients may feel better and score higher on function-oriented metrics but still not resume their prior level of athletic activity.^10^

While these improvements reached statistical significance, it is important to consider whether they translate into clinically meaningful changes. Only one of the included studies reported outcomes in terms of the minimal clinically important difference (MCID), finding that 87% of patients with FC-PVB–augmented meniscal repair achieved the MCID threshold of 11.1 points for the Lysholm score compared to 65.2% for the Tegner score's MCID of 0.9 points.^15,41^ This is consistent with the broader finding that more patients demonstrated improvements in Lysholm scores than in Tegner scores.^41^ None of the included studies evaluated the proportion of patients reaching the MCID of 10.9 points for the IKDC score.

In our review, fibrin clots were derived from either PVB or BMAC, both of which contain growth factors, including basic fibroblast growth factor (bFGF), transforming growth factor β (TGF-β), vascular endothelial growth factor, bone morphogenetic proteins, and stromal cell–derived factor 1 (SDF-1).^18,53,68^ However, FC-BMAC contains significantly higher levels of bFGF, TGF-β, hepatocyte growth factor, insulin-like growth factor 1, and SDF-1 compared to FC-PVB.^20,57^ These factors are known to enhance the viability and chondrogenic potential of meniscal fibrochondrocytes, particularly under hypoxic conditions typical of the meniscal environment,^40,49^ which suggests that FC-BMAC may be superior to FC-PVB in promoting meniscal repair.

This hypothesis may be supported by our findings in which none of the FC-PVB studies demonstrated significant improvements in Tegner scores while the FC-BMAC studies did. This suggests that FC-BMAC may play a role not only in functional recovery but also in facilitating a return to higher levels of physical activity. However, definitive conclusions cannot be drawn without direct head-to-head comparisons between FC-BMAC and FC-PVB in meniscal repair, and further studies with larger sample sizes are required to validate the potential benefits of FC-BMAC.

Similarly, Tegner activity scores did not show significant improvements in studies using PRF+PRP augmentation. However, both PRF alone and PRF+PRP, which are rich in growth factors believed to aid in meniscal healing, demonstrated meaningful gains in functional outcomes such as the IKDC, Lysholm, and combined Tegner-Lysholm scores, although none reported direct healing rates.

Beyond fibrin-based approaches, other biologic augmentation strategies such as PRP injections,^14,19^ stem cells,^13,56^ and marrow stimulation^1,16,35^ have also shown promise in enhancing meniscal healing.^1,16,35^ A systematic review and meta-analysis showed that PRP augmentation can reduce failure rates and improve pain and symptom scores compared to nonaugmented repair.^39^ However, improvements in functional metrics such as the IKDC or Lysholm score have not been consistently demonstrated.^39^

Importantly, each fibrin-based augmentation technique offers unique advantages and trade-offs. FC-PVB is simple, low cost, and minimally invasive, requiring no specialized equipment and allowing for intraoperative preparation.^6,27^ However, its biological potency may be limited by lower concentrations of growth factors, which may explain the lack of improvement in Tegner scores, despite gains in IKDC and Lysholm scores.^20^ FC-BMAC is biologically richer and may better support chondrogenesis and cell viability in hypoxic environments.^20^ This was reflected in improved functional outcomes, including Tegner activity scores, although the approach is more invasive and technically demanding because of marrow harvesting. PRF and PRF+PRP offer the sustained delivery of growth factors via a 3-dimensional fibrin matrix, meaning that there is natural cross-linking of fibrin fibers that trap platelets and leukocytes within a gel-like structure, helping to localize healing factors at the repair site while also providing a supportive framework for tissue regeneration.^31^ The PRF and PRF+PRP studies showed consistent improvements in clinical scores; however, these methods require centrifugation systems that may increase the cost^31^ (Table 5).

**Table 5:** Advantages and Disadvantages of Different Fibrin-Based Augmentation Techniques*^*a*^*

Type of Augmentation	Advantages	Disadvantages
FC-PVB	• Low cost • Minimally invasive • No need for specialized equipment • Easy intraoperative preparation	• Lower concentration of growth factors • No significant improvement in Tegner scores
FC-BMAC	• Higher biological potency because richer in growth factors	• Invasive bone marrow harvesting • Increased operative time and technical complexity
PRF and PRF+PRP	• Sustained release of healing factors • 3-dimensional fibrin scaffold	• Requirement of centrifugation equipment • Higher cost

a FC-BMAC, fibrin clot from bone marrow aspirate concentrate; FC-PVB, fibrin clot from peripheral venous blood; PRF, platelet-rich fibrin; PRP, platelet-rich plasma.

The included studies had few differences in fibrin clot preparation, delivery, and volume. For FC-PVB and PRF studies, the majority collected between 20 and 120 mL of autologous PVB and agitated it with a sterile instrument, such as a glass rod,^15,37,53^ stainless steel stick,^34^ or arthroscopy reamer,^33^ for 5 to 15 minutes until a clot formed, which was then rinsed, trimmed to match the tear, and delivered arthroscopically with a grasper or hemostat before being secured with repair sutures.^12,15,22,33,34,37,46,53^ The volume implanted was not standardized, and most studies tailored the clot size to the individual tear,^12,15,33,34,37,46,47,53^ while a minority reported approximate volumes of 1.5 to 2 mL^22^ or 1 to 2 mL.^66^ However, 2 studies diverged from autologous manual preparation, instead using commercial fibrin products: one used a purified 2-component fibrin glue system (Tisseel), injecting approximately 1 to 2 mL directly into the tear via a needle,^29^ and another used a commercial fibrin product (Tissucol) placed into the intrameniscal space before suture repair.^9^ Several technique articles have since described refinements to FC-PVB preparation beyond that used in the included studies, with the key innovations lying primarily in clot delivery and conditioning rather than preparation itself.^2,11,17,32,48,54,69^ Notably, 2 studies introduced shuttle-based delivery systems, using epidural needle–passed Prolene sutures and PDS sutures through a cannula, respectively, to allow more controlled and reproducible intra-articular placement compared to simple grasper insertion.^17,32^ Another study added iterative saline washing and gauze rolling to produce a firmer, more manipulable “fibrillar” clot, along with a sterile tube delivery system to prevent clot loss in the Hoffa fat pad, which is particularly advantageous for large, radial, or horizontal tears.^32^

For FC-BMAC, one included study prepared an autologous fibrin clot from approximately 50 mL of BMAC, which was stirred with a sintered glass rod for 8 to 10 minutes, shaped into a spindle construct with Vicryl suture tails, and delivered into the meniscal defect via a PDS suture through a 5-mm cannula under continuous irrigation before being secured with all-inside repair sutures.^20^ Building on this approach, one technique's key innovation was the addition of a polyglycolic acid sheet wrapping secured with 4-0 Vicryl, which improved intra-articular handling and reduced clot dissolution while providing the additional biological benefit of MSCs.^69^

This study has several notable strengths. First, it is the most comprehensive synthesis to date evaluating fibrin-based augmentation across all major biologic preparation methods, including FC-PVB, FC-BMAC, PRF, and PRF+PRP. By incorporating 15 studies with 461 menisci, this review provides the broadest overview of healing rates, complications, and functional outcomes across diverse tear patterns and avascular zones. Similarly, by comparing fibrin-augmented repair to nonaugmented repair (control) where available, our study contextualizes the magnitude of benefit conferred by fibrin-based scaffolds while also offering a biologically grounded explanation of these findings.

This study also has some limitations. First, there is heterogeneity in tear types, locations, and chronicity across the included studies, which may influence the healing potential and outcomes. Second, 2 studies (3 repair cases [1.5% of included repair cases]) did not explicitly define “partial healing,” which may limit comparability. However, most studies used similar arthroscopic or MRI-based criteria focused on tear closure and fluid signaling, supporting overall consistency in the healing assessment. Additionally, variability in surgeon experience and surgical techniques used for meniscal repair and augmentation further limits the generalizability and strength of the conclusions drawn. Lastly, only one randomized controlled trial directly compared fibrin-augmented repair to nonaugmented repair, highlighting the need for further high-quality comparative studies to better evaluate the potential added benefit of fibrin-based augmentation.

## Conclusion

Fibrin-based augmentation demonstrated high healing rates, low complication rates, and consistent improvements in clinical outcome scores after meniscal repair. Compared to nonaugmented repair, fibrin-augmented repair achieved superior healing and functional outcomes. These results may support the use of fibrin-based scaffolds as an effective strategy to enhance meniscal healing.

## Supplemental Material

sj-docx-1-ajs-10.1177_03635465261440395 – Supplemental material for Fibrin-Based Scaffold Augmentation During Meniscal Repair May Enhance Healing and Functional Outcomes: A Systematic Review Supplemental material, sj-docx-1-ajs-10.1177_03635465261440395 for Fibrin-Based Scaffold Augmentation During Meniscal Repair May Enhance Healing and Functional Outcomes: A Systematic Review by Khaled Skaik, Harjind Kahlon, Marc Daniel Bouchard, Prushoth Vivekanantha, Etienne L. Belzile, Bogdan A. Matache and Jihad Abouali in The American Journal of Sports Medicine
