# Supplementary material for: Fibrin-Based Scaffold Augmentation During Meniscal Repair May Enhance Healing and Functional Outcomes: A Systematic Review
Source: Am J Sports Med. 2026 Jun 3;54(12):3081–92. doi: 10.1177/03635465261440395 (PMC13620251; doi:10.1177/03635465261440395)
Supplement: Supplementary file 1 [file sj-docx-1-ajs-10.1177_03635465261440395.docx]

**Supplement S1.** MeSH terms and Boolean operators used to develop search strategy from PubMed, Embase, and OVID Medline.

*Database: PubMed <1946 to 2025 October 15>*

("Meniscus” OR "Meniscal Injuries"[Mesh] OR "Meniscal Repair"[Mesh] OR meniscus[tiab] OR menisci[tiab] OR meniscal[tiab] OR "meniscal tear*"[tiab] OR "meniscal repair*"[tiab] OR "meniscus repair*"[tiab]) AND ("Fibrin Tissue Adhesive"[Mesh] OR "Fibrin Glue"[Mesh] OR "Fibrinogen"[Mesh] OR "Platelet-Rich Fibrin"[Mesh] OR "Platelet-Rich Plasma"[Mesh] OR fibrin[tiab] OR "fibrin clot*"[tiab] OR "fibrin glue"[tiab] OR "fibrin sealant"[tiab] OR "platelet-rich fibrin"[tiab] OR "PRF"[tiab] OR "autologous fibrin clot"[tiab] OR "platelet fibrin matrix"[tiab] OR "exogenous fibrin clot"[tiab] OR "endogenous fibrin clot"[tiab] OR “Augmentation” OR “Platelet rich plasma”).

*Database: Embase <1974 to 2025 October 15>*

1. exp meniscal repair/. Results: 2688
2. fibrin glue/ or fibrin polymerization/ or fibrin clot/ or fibrin/. Results: 54709
3. knee meniscus/. Results: 15301
4. 1 and 2. Results: 84

*Database: OVID Medline <1964 to 2025 October 15>*

1. exp Meniscus/. Results: 9345
2. Fibrin/ or Platelet-Rich Fibrin/. Results: 17811
3. 1 and 2. Results: 27

**Supplement S2.** Quality assessment of included studies.

| **Study** | **Quality Assessment (JBI or MCMS)** |
| --- | --- |
| Davies et al. (2024) | JBI: 10/10 |
| Hashimoto et al. (2022) | JBI: 10/10 |
| Henning et al. (1991) | JBI: 7/10 |
| Ishimura et al. (1997) | JBI: 10/10 |
| Kale et al. (2022) | JBI: 9/10 |
| Kamimura et al. (2014) | JBI: 7/10 |
| Kemmochi et al. (2024) | JBI: 9/10 |
| Nakayama et al. (2020) | JBI: 9/10 |
| Nakayama et al. (2017) | JBI: 9/10 |
| Ra et al. (2013) | JBI: 8/10 |
| van Trommel et al. (1998) | JBI: 8/10 |
| Biedert et al. (2000) | MCMS: 79/100 |
| Chrysanthou et al. (2024) | MCMS: 83/100 |
| Kemmochi et al. (2018) | MCMS: 55/100 |
| Screpis et al. (2021) | MCMS: 65/100 |

MCMS: Modified Coleman Methodology Score. JBI: Johanna Briggs Institute.
